# Genome-wide patterns of copy number variation in the Chinese yak genome

**DOI:** 10.1186/s12864-016-2702-6

**Published:** 2016-05-20

**Authors:** Xiao Zhang, Kun Wang, Lizhong Wang, Yongzhi Yang, Zhengqiang Ni, Xiuyue Xie, Xuemin Shao, Jin Han, Dongshi Wan, Qiang Qiu

**Affiliations:** State Key Laboratory of Grassland Agroecosystem, College of Life Science, Lanzhou University, Lanzhou, 730000 China

**Keywords:** Yak, Copy number variation, Domestication, High altitude adaptation

## Abstract

**Background:**

Copy number variation (CNV) represents an important source of genetic divergence that can produce drastic phenotypic differences and may therefore be subject to selection during domestication and environmental adaptation. To investigate the evolutionary dynamics of CNV in the yak genome, we used a read depth approach to detect CNV based on genome resequencing data from 14 wild and 65 domestic yaks and determined CNV regions related to domestication and adaptations to high-altitude.

**Results:**

We identified 2,634 CNV regions (CNVRs) comprising a total of 153 megabases (5.7 % of the yak genome) and 3,879 overlapping annotated genes. Comparison between domestic and wild yak populations identified 121 potentially selected CNVRs, harboring genes related to neuronal development, reproduction, nutrition and energy metabolism. In addition, we found 85 CNVRs that are significantly different between domestic yak living in high- and low-altitude areas, including three genes related to hypoxia response and six related to immune defense. This analysis shows that genic CNVs may play an important role in phenotypic changes during yak domestication and adaptation to life at high-altitude.

**Conclusions:**

We present the first refined CNV map for yak along with comprehensive genomic analysis of yak CNV. Our results provide new insights into the genetic basis of yak domestication and adaptation to living in a high-altitude environment, as well as a valuable genetic resource that will facilitate future CNV association studies of important traits in yak and other bovid species.

**Electronic supplementary material:**

The online version of this article (doi:10.1186/s12864-016-2702-6) contains supplementary material, which is available to authorized users.

## Background

Copy number variations (CNVs), a form of genomic structural variation, are defined as duplications or deletions of DNA fragments that range in size from at least 50 base-pairs (bp) to more than several megabase-pairs (Mb), causing a different copy number of specific genomic regions among individuals of a species [1–4]. CNV represents an important source of genetic variation complementary to SNP data, but which affects a higher percentage of genomic sequences and has potentially stronger effects on phenotypic diversity and evolutionary adaptation, through changing gene dosage and transcript structure, and regulating gene expression and function [5].

As a common feature of vertebrate genomes, the foundational studies of CNV have been conducted in humans. Around 2,057,368 CNVs that correspond to over 24,032 CNV regions have been identified in humans and these may account for >9.5 % of the human genome, with over 12 % of gene sequences involved in CNV regions [6]. CNVs that underlie complex traits such as obesity, diabetes, Alzheimer’s disease and Autism spectrum disorders have been detected in human patients [5]. In recent years, advances in genomic technologies have made it increasingly feasible to screen comprehensively for CNVs, and so interest in CNV detection has extended to livestock species, with considerable advances being made [7–9]. CNV maps have been constructed for cattle [10], horse [3], goat [11], sheep [12], pig [13], dog [14] and chicken [15], providing a very valuable resource for evolution and genetic improvement research in livestock. In addition, there is growing evidence for CNVs associated with phenotypic variation, disease susceptibility, environmental adaptation and production traits in livestock species. For example, the copy number variation of the *AMY2B* gene probably allowed dogs to thrive on a relatively starch-rich diet during their domestication [16]. The *ASIP* CNV allele is almost entirely associated with different coat colors in different goat breeds [17] and in Tibetan sheep [18]. The most recognizable chicken pea-comb phenotype is attributed to a duplication near the *SOX5* gene [19]. A CNV involving the *AKR1C* gene is considered possibly responsible for testicular androgen synthesis and sexual development in horse [3]. The DNA dosage and EST expression of CNVs overlapping with the *Ntn1* gene may influence meat quality in pigs [20]. In bovid species, several important genes related to clear phenotypic changes and breed differences have been modified by CNVs: the copy number variations of the *HSFY* and *ZNF280BY* genes are associated with male reproductive traits in Holstein bulls [21]; *PLA2G2D* located in a CNV region is associated with aspects of body size in Chinese bulls such as heart girth and hucklebone width [22]; hereditary myopathy of diaphragmatic muscles in Holstein-Friesian cattle has been linked to deletion of the *HSPA1B* gene [23]; and the *APOL3* gene involved in lipid transport is highly duplicated in beef breeds [24]. These studies reveal that many beneficial CNVs may have been artificially selected in livestock during domestication and could be associated with or affect important traits of economic interest. However, only a few studies provide a comprehensive characterization of the evolutionary impact of CNVs comparing a wild and domestic population.

The yak (*Bos grunniens*) is the only major livestock that can survive the extremely cold, harsh and oxygen-poor conditions and take full advantage of the limited grassland resources on the Qinghai-Tibetan Plateau (QTP) [25]. Yak were domesticated by the early nomadic people from wild yak more than 7,300 years ago [26]. Nowadays, there are more than 14 million domestic yaks, providing necessities for Tibetans and other nomadic pastoralists in high-altitude environments; in addition, there are still 15–20 thousand wild yaks roaming the northwestern QTP [27]. It should be noted that the domestic yak is the only large animal that still coexists with its wild ancestors in a similar environment, as wild progenitors of other domestic livestock are now extinct or geographically dispersed [25, 28]. Therefore, the wild and domestic yak provide a good framework for studying effects of CNV in large livestock domestication [29]. Indeed, a more comprehensive understanding of CNVs at the whole-genome level could provide additional evidence for unraveling the genetic basis of yak domestication. However, as far as we know, there is only one published dataset reporting only 161 CNV regions based on just two yak individuals using the cattle-specific Nimblegen3x720K CGH array [22].

Although comparative genomic hybridization (CGH) and SNP arrays are routinely used for CNV identification, the performance of these methods are heavily depending on the marker density and the specially designed non-polymorphic probes [30–32]. The advent of next-generation sequencing (NGS) technologies and complementary analysis has provided innovative approaches to systematically screen CNVs at a whole-genome level, especially in non-model organisms [3, 30, 33]. Among different analysis programs to detect CNVs using sequence data, the read depth (RD) method have become a major approach due to its stronger ability to estimate the exact copy numbers and identify CNVs in complex genomic regions [33, 34]. Thus, the recent completion of the yak genome and population genome studies now allow for a comprehensive screening of CNV [26, 35, 36].

Here we describe the first genome-wide and systematic analysis of CNVs in yak using NGS genome re-sequencing data from wild and domestic yak. This CNV map was constructed for three main reasons. First, to develop and enrich new genomic variations in order to facilitate further research on yak and other bovid species and make it available to the scientific community. Second, to determine genes affected by CNV that differ between wild and domestic yak, and evaluate whether extensive CNV is related to domestication. Third, to compare the CNVs in individuals from different altitudes and assess the evolutionary impact of CNVs in adaptations to life at high altitude.

## Results and discussion

### CNV discovery and data set statistics

**Fig. 1:**
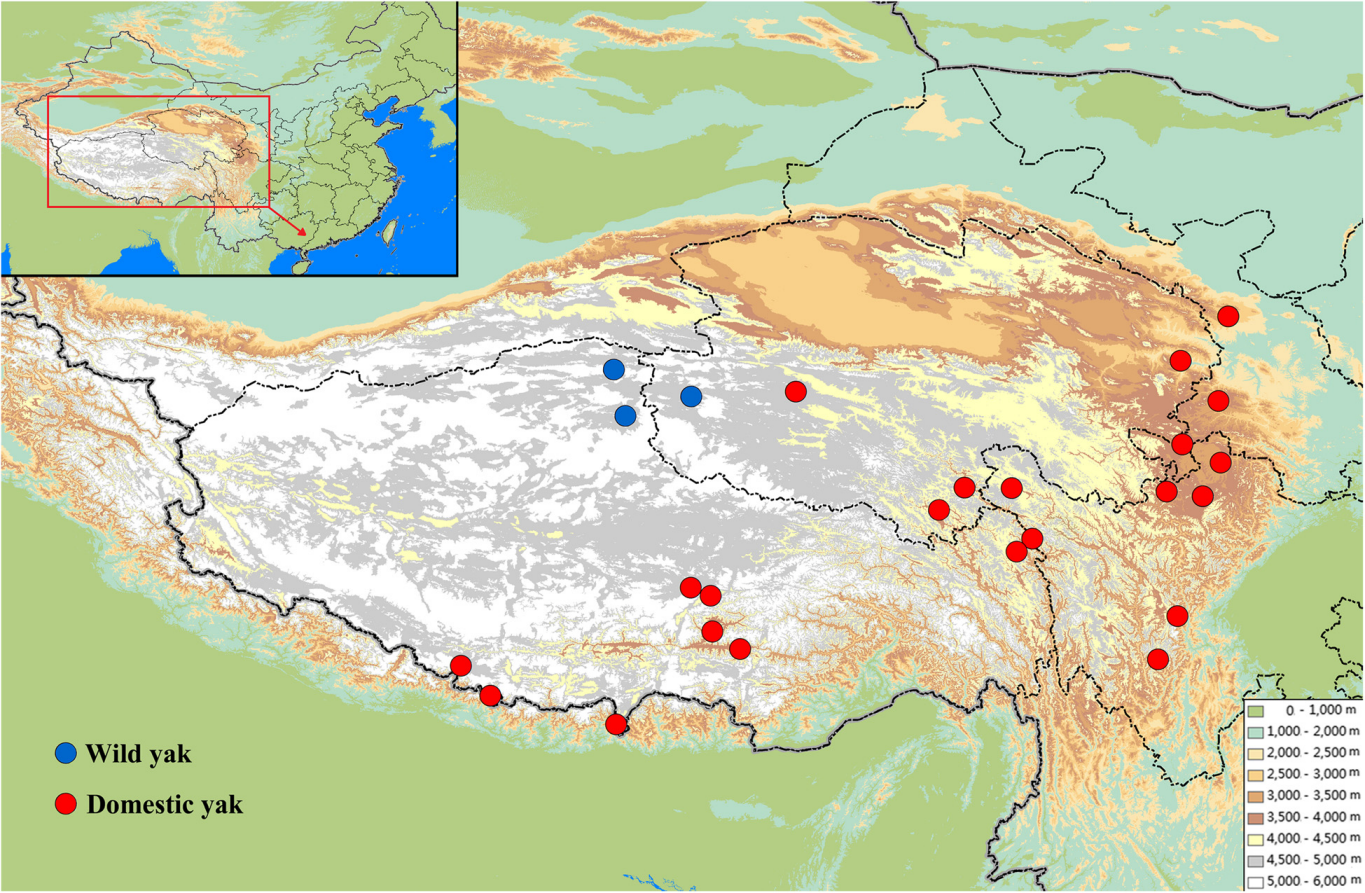
Geographical distribution of the yak samples used in this study. The sampling locations and altitudes for wild (*blue*) and domestic (*red*) yak were mapped using ArcGIS software. Geographic (ESRI: www.esri.com) and altitude (WorldClim: www.worldclim.org) parameters were obtained from freely accessible data layers

We used whole genome re-sequenced data from our previous study, based on 14 wild and 65 domestic yaks from widely spaced locations across the QTP (Fig. 1), representing three highly diverged mitochondrial lineages and broad genetic diversity [28]. A total of 1.56 Tb of sequences with an average depth of 6.7× were used (Additional file 1) [26]. Mapping these reads to the yak reference genome [35] revealed that at least 93 % of the genome was covered by reads from a single individual, and, on average, 98 % of the reference genome was covered, indicating that the data are sufficient and of high enough quality for CNV detection [37].

**Fig. 2:**
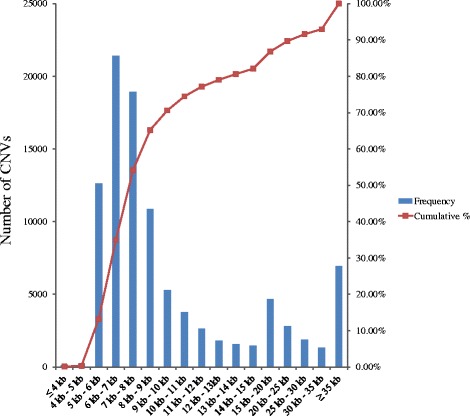
CNV size interval distribution. Average CNV size is 15.6 kb and the median size is 8.0 kb

Using the CNVnator software based on the RD method [37], we detected a total of 98,441 CNV events from the 79 individuals (Fig. 2 and Additional file 2). The average number of CNVs per individual was 1,246 (ranging from 954 to 2,952) with an average of 291 gain and 955 loss events. The size of the CNVs identified varied from 1.5 kb to 1,226.0 kb with an average size of 15.6 kb and a median size of 8.0 kb. Specifically, only 1.8 % of all CNV calls were present in a single individual (Additional file 3). The results of CNVs identified and the location information for each individual are listed in Additional file 2.

**Fig. 3:**
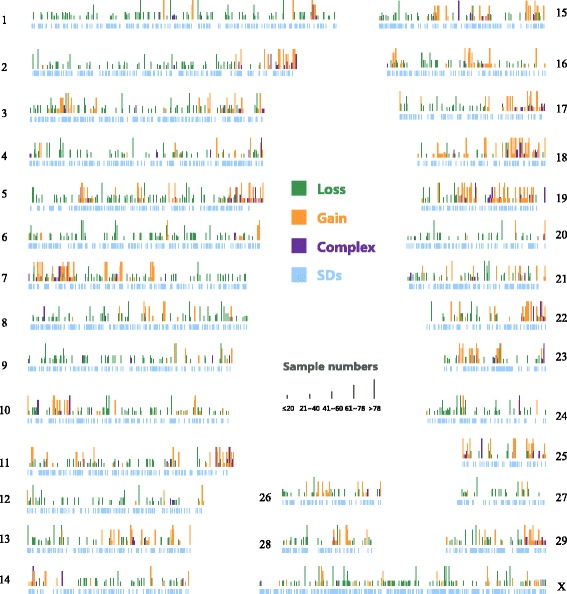
Genomic landscape of yak copy number variation regions and segmental duplications. The SDs are plotted as blue bars. The CNVRs are illustrated above the SDs in green (loss), orange (gain) and purple (complex of gains and losses). The chromosome numbers (1–29, X) are shown nearby correspond CNV landscape, and the bar height represents different sample numbers (≤20, 21–40, 41–60, 61–78, > 78 )

We further defined 2,634 regions with copy number variations (termed CNVRs) by merging all overlapping calls across multiple individuals into unique regions and filtering the ones that were present in fewer than four individuals. These CNVRs occupied a total of 153 Mb or 5.7 % of the yak genome, substantially more than the recent study in two yak individuals based on a CGH array approach (33 Mb, 1.25 %) [38]. The length of the CNVRs varied from 3 kb to 1,309 kb with an average of 58 kb. Among all CNVRs, 234 (8.9 %) were found to be common to all 79 individuals (Fig. 3 and Additional file 4). According to the type of CNVRs, they were divided into three categories, including 785 gain, 1,575 loss and 274 both (gain and loss within the same regions from different individuals) events (Fig. 3). Although it is difficult to compare the CNVRs in different studies due to the different diversity of individuals and the technology used for detection, our study based on next-generation sequencing, using 79 different wild as well as domestic yaks resulted in better resolution and higher confidence in calling CNVRs than past research [22]. Thus, most of the CNVRs discovered in this study are novel compared to previous studies and they represent the largest catalog of yak specific CNVRs to date.

We evaluated the accuracy of individual copy number variations predicted by performing a series of real time-polymerase chain reactions (qPCR). The experimental validation showed that 88 % of the CNVs (56/64) had an accurate copy number and 100 % were in agreement with the predicted patterns (Additional file 5). It should be emphasized that not all true CNVs could be detected by qPCR, especially some low-copy duplications with lower sequence similarities. Thus, a 12 % false positive rate is a conservative estimate in our CNV analysis, representing a relatively low false positive rate in our CNV calling and CNVR definitions [24, 39, 40]. We further estimated the accuracy using 6 deep-coverage (30×) yak genomes, including three domestic and three wild individuals, revealing that 85 % of our original CNV calls were supported by higher coverage genome data. Moreover, considering that the quality of the assembled reference as well as the annotated repeats is crucial to discovering CNVs using the RD method, additional experimental validation, such as qPCR from more individuals, array comparative genomic hybridization (aCGH) and fluorescent in situ hybridization (FISH), will be required to obtain more accurate information about the CNVs and to exclude false positives [3, 24].

### SD detection and comparison with CNVRs

Segmental duplications (SDs) were shown to be one of the catalysts and hotspots for CNV formation in many species [3, 24, 41], and we tested whether the non-random association between CNVs and SDs was preserved in yak. Based on whole-genome assembly comparison (WGAC) [42] and whole-genome shotgun sequence detection (WSSD) [43] methods, we first identified 47,740 and 5,381 putative segment duplication events, respectively. By merging these two data sets, we finally predicted 50,800 segments, spanning 113.8 Mb of DNA sequence and comprising 4.3 % of the yak genome (Additional file 6), a similar level to that of the bovine genome (3.11 %) [44]. We subsequently compared the overlap between the SDs identified above and the CNVRs and found that 56 % of large CNVRs (>15 kb) directly overlapped with SDs. Random simulations further confirmed significant relationships between 5 Mb flanking regions of CNVRs and their overlap with SDs (Additional file 7), consistent with the previously studied CNVs that were enriched with SDs [10].

### Gene content of CNV regions

Among the 2,634 CNVRs described above, 961 (36.5 %) harbored a total of 3,879 protein-coding genes according to the current yak genome (Additional file 8). These represent a valuable resource for future studies on the relationship between CNV genes and phenotypic variation. Gene ontology (GO) enrichment analysis indicated that CNVRs harbored genes that were mainly involved in sensory perception and olfactory receptor activity (Additional file 9), such as the GO categories “olfactory receptor activity”, “sensory perception”, “sensory perception of chemical stimulus” and “sensory perception of smell”, which was consistent with the observation that olfactory gene families are large and associated with CNVs [10, 13, 39]. In addition, gene families involved in sensory perception are usually fast evolving due to their importance in the organism responding to rapid changes in the environment and they have been repeatedly detected in CNV regions of several livestock genomes. Our previous comparative genome study also found that gene families related to sensory perception were substantially expanded in yak compared to other mammals [35].

### Population-differentiated CNVRs between wild and domestic yak

To reveal the potential contributions of CNVs in the process of yak domestication, we carried out a comparative study to find the CNVRs with significantly different copy number between domestic and wild yak. We identified 121 CNVRs potentially selected during domestication, containing 100 annotated protein coding genes (Additional file 10).

Strong selection on reducing aggressive behavior and neurological traits is often involved in the initial step of animal domestication [45], and our previous domestication selective sweep analysis based on SNP data identified 38 genes related to these functions [26]. In this study, we found eight more genes with high CN differentiation between domestic and wild yak that are involved in neuronal development and are associated with behavior, further indicating that genes related to brain and nervous system development might underlie the processes that led to the successful domestication of the yak. For example, *GRIN2D* is related to normal brain development and associated with learning, working memory and behavioral attention [46]; *FTL* encodes the ferritin protein and mutations in this gene could lead to behavioral abnormalities and cognitive impairment [47]; *NTN5* is highly expressed in neurogenic regions of the adult brain and controls neurogenesis [48]; *SHANK3* plays a role in synapse formation and dendritic spine maturation [49]; *KCNJ14* has a function in controlling the excitability of motor neurons [50]; *CA11* is abundantly expressed in the brain and has a general role in the central nervous system [51]; *NTF4* encodes a neurotrophic factor which controls neuron differentiation [52]; and *ARSA* is related to many neurodegenerative diseases [53].

Another important consequence of livestock domestication is a change in their reproductive efficiency, such as age of puberty, sperm production, ovulation rate and embryonic mortality [54]. We found seven CNV-associated genes involved in reproductive performance traits. *TSEG2*, *AKR1C3* and *IZUMO1* are sperm-specific expressed genes and have a role in spermatogenic cell development and fertility [55–57]. *LHB* is expressed in the pituitary gland and promotes spermatogenesis and ovulation; mutations in this gene are associated with polycystic ovary syndrome in women [58]. *SPACA4* encoded protein is retained on the inner acrosomal membrane of spermatozoa and plays a role in sperm–egg binding and fertilization [59]. *FGF21* encodes fibroblast growth factor protein, which is involved in embryonic development [60]. *DMBT1* encoding lactoprotein as a scavenger receptor protein in milk, which can suppress various bacterial infections in vitro and plays an important role in the innate immunity of breast-fed infants [61].

Previous studies have revealed that domestication leads to relaxation of selective constraints in the yak mitochondrial genome, because the domestic yak and wild yak have different energy demands and metabolic efficiency [62]. We found four genes, *ND4L* [63], *SCO2* [64], *CO1* [65] and *ND1* [66], related to mitochondrial oxidative phosphorylation (OXPHOS) that exhibit marked CN variation between wild and domestic yak, indicating that CNV may affect genes involved in energy metabolism during yak domestication.

**Fig. 4:**
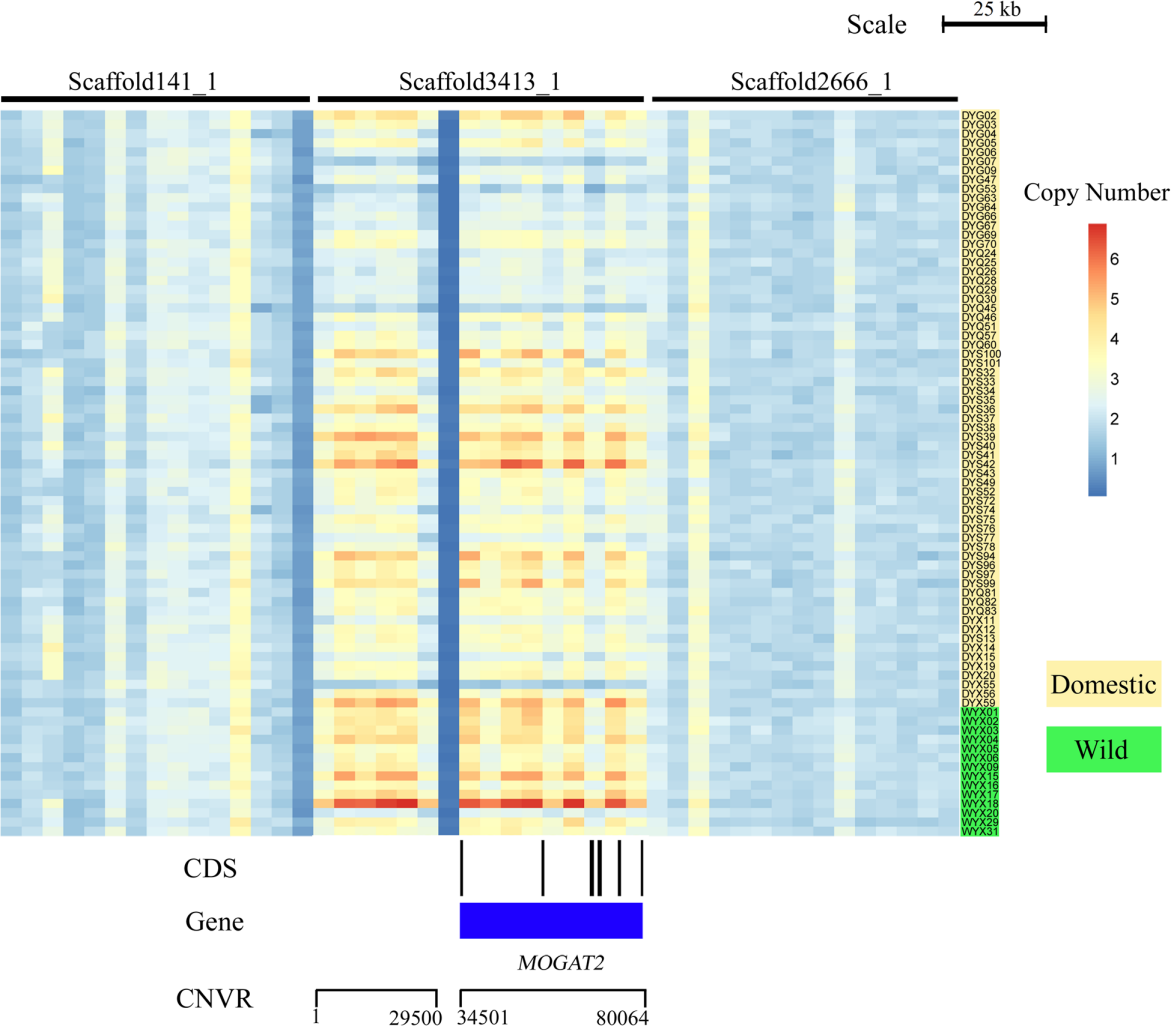
Heatmap of CNVR with *MOGAT2* gene located in scaffold3413_1. Average read depths were plotted every 5 kb of the genome. The CNVR with the *MOGAT2* gene represents different average normalized read depths of a specific region. The domestic yak (*yellow*) exhibited a lower copy number than the wild ones (*green*). CDS and gene are shown at the bottom (*black rectangle*, coding sequence; *blue box*, whole gene)

The progress of domestication of livestock would also affect nutritional uptake, absorption and metabolism [67]. We found that seven interesting CNVRs harbored genes related to nutrition metabolism and there were significant differences in copy number between domestic and wild yak. For instance, *MOGAT2* [68], *GYS1* [66] and *DHDH* [69] are involved in the metabolism of sugars (Fig. 4); *HSD17B14* [70] and *CPT1B* [71] are involved in lipid metabolism; *BCAT2* is involved in amino acid metabolism [72]; and *MIOX* is involved in carbohydrate metabolism [73]. In addition, we identified four genes (*CHRM3* [74], *KLF6* [75], *GPC1* [76] and *CHKB* [77]) related to meat production and quality, which is also an economically significant trait that has been extensively considered during the artificial selection process.

Taken together, our results suggest that some CNVRs may have been under selection pressure during yak domestication and these are associated with behavior, physical characteristics and economically significant traits. Consequently, additional functional experiments are needed to verify the contributions of the identified genes located in CNVRs to domestication.

### Population-differentiated CNVRs between yak living at high and low altitudes

**Fig. 5:**
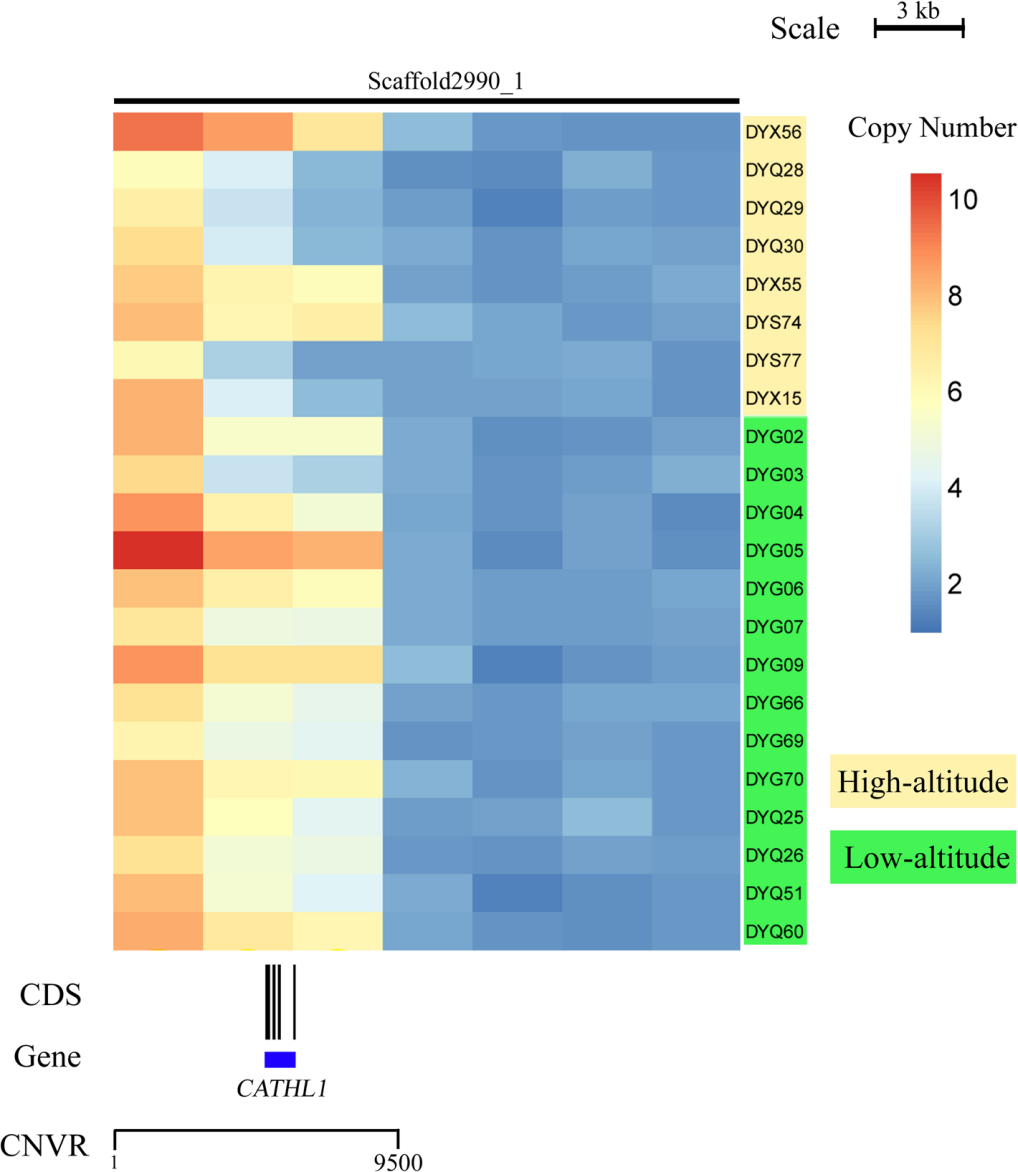
Heatmap of CNVR with *CATHL1* gene located in scaffold2990_1. Average read depths were plotted every 3 kb of the genome. The CNVRs with the *CATHL1* gene represents different average normalized read depths of a specific region. The yaks living in low-altitude (*yellow*) exhibited a lower copy number than the ones living in high-altitude (*green*). CDS and gene are shown at the bottom (black rectangle, coding sequence; blue box, whole gene)

In order to understand better the evolutionary impact of CNVs in adaptations to high altitude, we compared the CNVs between domestic yak living in high-altitude (>4,000 m) and low-altitude (<2,500 m) areas. We found 85 CNVRs that are significantly different between these two groups: 29 of them harbor 41 protein-coding genes (Additional file 11). First, compared to the low-altitude population, yak living at high-altitudes must overcome the severe environmental challenges of hypoxia and an increased risk of high-altitude illness, such as pulmonary arterial hypertension and emphysema. We identified three genes involved in the response to hypoxia – *MRP4* [78], *DCC* [79] and *DEXI* [80] – for which the copy number differed between high and low-altitude domestic yak. The *MRP4* gene encodes ATP-binding cassette (ABC) transporters, regulating intracellular levels of cAMP and cGMP in arterial smooth muscle cells; inhibition of *MRP4* expression can prevent and reverse hypoxia-induced pulmonary arterial hypertension [78]. The *DCC* gene encodes the Netrin 1 receptor, which plays an important mediating function in protecting against hypoxia-induced mitochondrial apoptosis [79]. *DEXI* expression was increased in emphysema tissues and is associated with disease progression [80]. Second, we found six CNV genes related to the immune system that may be involved in high altitude adaptation. *ULBP17* encodes the immune system-activating receptor on T-cells and is related to pathogen- and parasite-resistance [24]. *CIITA* is a positive regulator of class II major histocompatibility and mutations in this gene are associated with diseases of the immune system [81]. *CATHL1* encodes cathelicidin, which is important in innate immune defense against bacterial infection (Fig. 5) [82], *BoLA-DQA2, BoLA-DQA3* and *BoLA-DQB* are key leukocyte antigen genes associated with the immune response. These CNVRs that span potential genes influencing the immune system may reflect the substantially different diseases triggered by the parasites and arbovirus at high and low altitudes [83–85]. These results suggest that CNV is an important type of genetic variation that may play a role in yak adaptation to high-altitude environments.

## Conclusions

We performed genome-wide CNV detection based on whole genome sequencing data for 14 wild and 65 domestic yaks. A total of 2,634 CNVRs comprising 153 megabases of the yak genome were identified; this represents the largest source of CNVs identified and the highest-resolution individualized CNV map constructed for the yak genome to date. The inferred CNVRs contain 3,879 functionally annotated genes and further functional analyses reveal CNVRs enriched for genes related to sensory perception and responses to stimuli. Comparing the different CNVs in wild and domestic yak suggests that many genes overlapping with CNVRs play a role in behavior, physical characteristics and economically significant traits. In addition, some novel genes, including *DEXI, DCC*, and *MRP4*, were found to be covered by CNVRs related to adaptation to high-altitude environments. Our study represents a comprehensive genomic analysis of CNVs in yak and provides valuable insights into the evolutionary dynamics of copy number variation, in the context of domestication and high-altitude adaptation. The database presented in this study will provide an important genetic resource for future work on phenotypic variation and breeding in both yak and other bovid species.

## Methods

### Data sets

The whole genome sequencing data were obtained from our previously study (Additional file 1, which were deposited in the European Molecular Biology Laboratory (EMBL/EBI) Nucleotide Sequence Database under the accession number PRJNA285834). Briefly, the muscle samples were obtained from 14 wild yaks and 65 domestic yaks of diverse breeds from widely spaced locations across the QTP. DNA was extracted using a standard phenol/chloroform extraction method; paired-end sequencing libraries with an insert size of 500 bp were sequenced using an Illumina HiSeq 2000 platform.

### Sequence quality checking and read alignment

Using a custom Perl script, we filtered out the low quality reads based on the following criteria: (i) reads with ≥10 % unidentified nucleotides (N); (ii) reads for which more than 65 % of the read length had a Phred quality value ≤7; (iii) reads with more than 10 bp aligned to the adapter, allowing 2 bp mismatches; and (iv) duplicate reads. Reads were also trimmed if they had three consecutive base pairs with a Phred quality value of 13 or below, and discarded if they were shorter than 45 bp. The pair-end sequence reads were mapped to the *B. grunniens* reference genome using BWA-MEM (0.7.10-r789) with default parameters. SAMTOOLS [86] was used to sort and index the resulting Binary Alignment Map (BAM) format files. In order to reduce the inaccuracy alignment, the Genome Analysis Toolkit [87] was used to realign reads located in regions around indels [36] after assigning read group information pertaining to library, lane and sample identity in the *Picard* software (http://broadinstitute.github.io/picard/, version 1.92).

### CNV detection and CNVR definition

CNVs were detected through read-depth analysis, in which the number of copies presented is inferred from sequence depth of whole genome sequencing data [13]. By combining the established mean-shift approach, multiple-bandwidth partitioning and GC correction, CNVnator was used to detect CNVs. It has been suggested that this approach is superior to other methods with respect to accuracy of the copy number estimate and the precision of sensitivity and specificity [34]. For each individual, the realigned BAM file was processed in CNVnator. Following the authors’ recommendations, we choose the optimal parameters for different individuals, and trimmed the boundaries of CNVs on the basis of 500-bp bins to avoid bias caused by different coverage. The fraction of reads that can map to two or more locations was denoted q0. All CNV calls with q0 >50 % were filtered out, followed by any remaining CNV events whose RD significantly differed from the average genome RD (t-test corrected for multiple hypotheses testing; *p* < 0.05). Previous studies have shown that RD based analysis have reduced power and reliability to detect small CNVs events [37, 88]. To reduce the false positive discovery rate and avoid misinterpreting the results, we only retained the CNVs longer than 1.5 kb for further analysis [13, 39]. In consideration of the fact that the yak is diploid, to reflect homozygous and heterozygous deletions, we normalized the copy number to 2.

The copy number variation region (CNVR) is defined as a combined region of overlapping CNVs on the genome. CNVRs are merged from different samples with any amount of overlap by extending the boundaries of the overlapping CNVs [89]. Here, all the CNVRs were defined using a custom Perl script. To reduce the false positive discovery rate further, only the CNVRs present in more than four samples were used for functional and comparative analysis, thus minimizing the bias caused by uniformity of sequencing coverage.

### qPCR and high depth resequencing validation

To evaluate the accuracy of our copy number assignments, we randomly selected eight gain and eight loss genic CNVRs. Thirty-two different individuals were randomly selected to validate these CNVRs by quantitative PCR (qPCR). A segment of bovine basic transcription factor 3 (*BTF3*) gene was chosen as a reference location for all qPCR experiments, because neither CNVs nor SDs overlapping this gene were found in our dataset nor in any previous studies of bovid species [24, 90]. Primers for qPCR validation were designed using the Primer3 webtool (http://frodo.wi.mit.edu/). The amplicon length was set to 100–200 bp, and the regions with GC percentage were between 30 and 60 % (Additional file 5).

Genomic DNA was extracted and purified from tissue using the standard phenol–chloroform method [91]. Using the CFX96 Real-Time PCR System (BIO-RAD), we performed the qPCR experiments in a total reaction volume of 20 μl, which contained 50 ng of template DNA, 10 mM primers and the reagents in the SYBR Green PCR kit (Takara). The copy number differences were determined using a standard ΔΔCt method and compared to the diploid reference gene *BTF3* as previously described [24].

We also used a high-depth (30×) re-sequencing dataset based on three domestic and three wild yaks to estimate the accuracy of our original identified CNV events. We calculated the coefficient of variation (cv%) between the high-depth-sequencing predicted and our original predicted copy numbers. The difference was considered to be acceptable if cv % was <0.25.

### Segmental duplication detection and association with CNV distribution

Using the yak_1.1 yak genome assembly, we applied two well-established computational approaches, whole-genome shotgun sequence detection (WSSD) and whole-genome assembly comparison (WGAC) to detect putative segmental duplications. Briefly, WGAC identifies paralogous sequences >1 kb in length with >90 % sequence identity, and WSSD identifies genomic regions that exhibit significant depth of coverage by aligning whole-genome shotgun sequencing reads to the reference genome sequence. The final SD dataset was obtained by merging these two results. Association between CNVs and SDs was tested by random simulations using a custom Perl script as previously published [10].

### Gene content and gene ontology

By searching for the coordinate of each CNVR in the yak genome assembly and gene annotation, we assessed the gene contents of each CNVR. Only the genes that were primarily comprised of CNVRs (>50 %) were retained. Functional classification of GO categories was performed using the Blast2GO program [92]. Enrichment analysis was performed and the hypergeometric test was used to calculate the statistical significance of enrichment. The P values were adjusted by FDR and the adjusted P value cut-off was 0.05. GO terms associated with the CNVRs and whole genome background were plotted by WEGO (http://wego.genomics.org.cn/cgi-bin/wego/index.pl).

### Comparison between different populations

We carried out two comparative studies to identify CNV genes with high differentiation between populations. One was domestication related, including 65 domestic and 14 wild samples; the other was high-altitude adaptation related, only including domestic yak living at high (>4,000 m, eight samples) and low-altitude (<2,500 m, 14 samples) areas. With the aim of identifying population-differentiated CNVRs more accurately, two statistical measures were used during comparisons: a metric analogous to *F*_ST_ (the fixation index) named *V*_ST_ [93] and the student’s t-test. For each locus, the *V*_ST_ was calculated using the formula *V*_ST_ = (*V*_T_–*V*_S_)/*V*_T_ as previously published. Here, *V*_T_ is the total variance in CN between the two populations and *V*_S_ is the average of the variance within each population, weighted for its sample size [39]. The distributions of CNs between different groups were also compared using the student’s t-test. Only loci exhibiting significantly different CNs based on these two statistics (top 5 % of all the *V*_ST_ values and P-value < 0.05) were considered to be population-differentiated CNVs, representing a rigorous criteria to identify differential CNVRs between populations. All these statistics were calculated using the R package.

### Heatmap analysis

We performed heatmap analyses based on the depth of each sample. For each individual, the depth of every base was computed using the “depth” command in SAMTOOLS [86]. As the estimated copy number, the ratio of the average depth of each window to the effective depth of each individual was then calculated. The pheatmap R package was used to plot these effective copy number values for all individuals.

### Ethics statement

All animal specimens were collected legally. Animal collection and utility protocols were approved by the Ethics Committee of Animal Experiments at Lanzhou University and in accordance with guidelines from the China Council on Animal Care.

### Consent for publication

Not applicable.

### Availability of data and material

The datasets supporting the conclusions of this article are available in the European Molecular Biology Laboratory (EMBL/EBI) Nucleotide Sequence Database repository (accession code PRJNA285834). Other supporting data are included as Additional files: 1, 2, 3, 4, 5, 6, 7, 8, 9, 10 and 11.

## Additional files

Additional file 1: Basic information relating to yak used in this study, their types, geographical distributions and resequencing data used in the CNV analysis. (XLS 45 kb) Additional file 2: List of all 98,441 CNV events in this study. (XLSX 2719 kb) Additional file 3: Summary of CNV events in different individuals. (XLS 35 kb) Additional file 4: List of all CNVRs in the yak genome. (XLSX 784 kb) Additional file 5: Primer sequences and confirmation results for qPCR. (XLS 39 kb) Additional file 6: Statistics for yak segmental duplication. (XLS 28 kb) Additional file 7: Relationships between flanking distances and numbers of yak CNVRs overlapping with SDs. The observed overlaps between CNVRs and SDs are plotted as little rings; the results of every 5000× random simulations are shown as filled dots with error bar. (PNG 152 kb) Additional file 8: Gene ontology annotations for genes covered by whole genome and CNVRs. The left y-axis indicates the percentage of a specific category of genes in a main category, while the right axis indicates the number of genes in it. (PNG 1006 kb) Additional file 9: Over-representation / under-representation of molecular function, cellular component and biological process terms. (XLS 30 kb) Additional file 10: Population-differentiated CNVRs with annotated protein coding genes when comparing wild and domestic yak. (XLS 40 kb) Additional file 11: Population-differentiated CNVRs with annotated protein coding genes when comparing yak living at high and low altitudes. (XLS 35 kb)

## Abbreviations

ABC: ATP-binding cassette
CGH: comparative genomic hybridization
CN: copy number
CNV: copy number variation
CNVR: region of copy number variations
GO: gene ontology
MHC: major histocompatibility complex
NGS: next generation sequencing
OXPHOS: oxidative phosphorylation
qPCR: real time-polymerase chain reactions
QTP: Qinghai-Tibetan Plateau
RD: read depth
SD: segmental duplication
WGAC: whole-genome assembly comparison
WSSD: whole-genome shotgun sequence detection
