# Supplementary figures and images for: Genome-wide patterns of copy number variation in the Chinese yak genome
Source: BMC Genomics. 2016 May 20;17:379. doi: 10.1186/s12864-016-2702-6 (PMC4875690; doi:10.1186/s12864-016-2702-6)

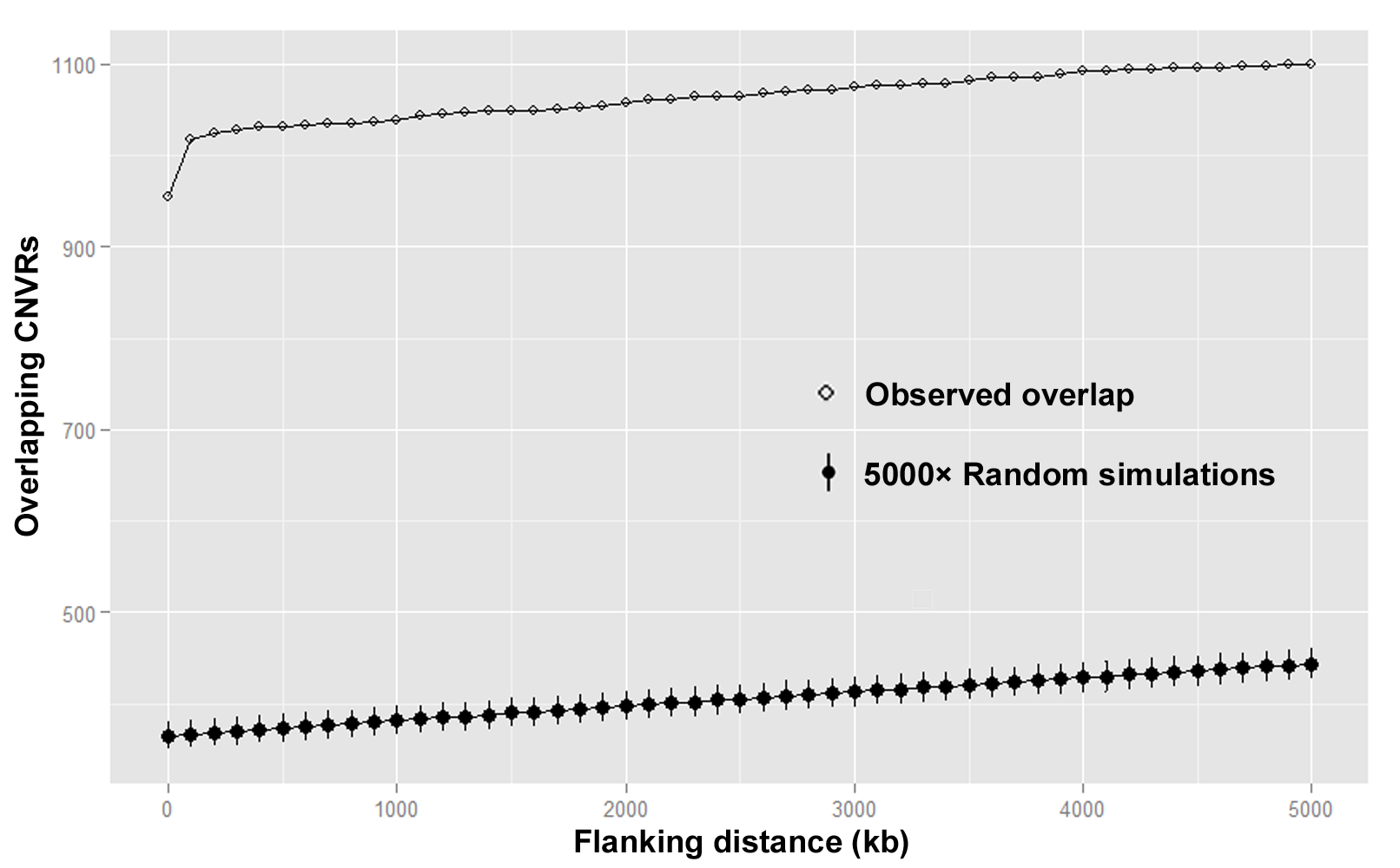

Supplement: Additional file 7: — Relationships between flanking distances and numbers of yak CNVRs overlapping with SDs. The observed overlaps between CNVRs and SDs are plotted as little rings; the results of every 5000× random simulations are shown as filled dots with error bar. (PNG 152 kb) [file 12864_2016_2702_MOESM7_ESM.png]

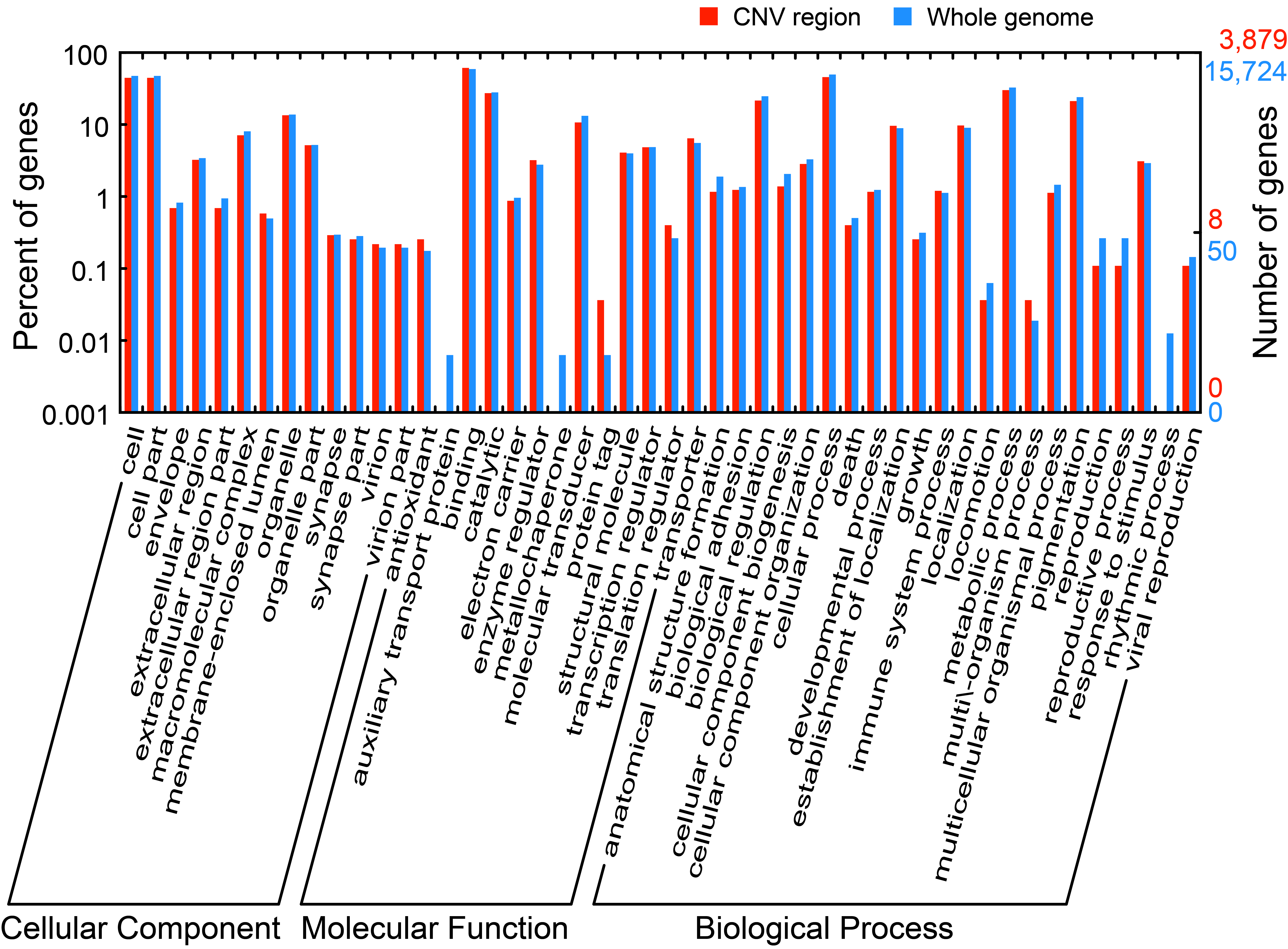

Supplement: Additional file 8: — Gene ontology annotations for genes covered by whole genome and CNVRs. The left y-axis indicates the percentage of a specific category of genes in a main category, while the right axis indicates the number of genes in it. (PNG 1006 kb) [file 12864_2016_2702_MOESM8_ESM.png]
